# Combining green cards, telephone calls and postcards into an intervention algorithm to reduce suicide reattempt (AlgoS): P-hoc analyses of an inconclusive randomized controlled trial

**DOI:** 10.1371/journal.pone.0210778

**Published:** 2019-02-01

**Authors:** Antoine Messiah, Charles-Edouard Notredame, Anne-Laure Demarty, Stéphane Duhem, Guillaume Vaiva, on behalf of AlgoS investigators

**Affiliations:** 1 INSERM Research unit U-1178 “Mental Health and Public Health”, Centre de recherche en Épidémiologie et Santé des Populations (CESP), INSERM, Université Paris-Sud, UVSQ, Université Paris-Saclay, Villejuif, France; 2 SCALab Laboratory, CNRS, UMR 9193, Universités de Lille, Lille, France; 3 Department of Child and Adolescent Psychiatry, Hôpital Fontan, CHRU Lille, Lille, France; 4 Inserm Clinical Investigation Center (CIC) 1403, CHRU and Universités de Lille, Lille, France; 5 Department of Adult Psychiatry, Hôpital Fontan, CHRU Lille, Lille, France; VA Medical Center, UNITED STATES

## Abstract

**Background:**

Brief contact interventions (BCIs) might be reliable suicide prevention strategies. BCI efficacy trials, however, gave equivocal results. AlgoS trial is a composite BCI that yielded inconclusive results when analyzed with Intention-To-Treat strategy. In order to elicit intervention strengths and weaknesses, post-hoc analyses of AlgoS data were performed.

**Methods:**

AlgoS was a randomized controlled trial conducted in 23 French hospitals. Suicide attempters were randomly assigned to either the intervention group (AlgoS) or the control group (Treatment as usual TAU). In the AlgoS arm, first-time suicide attempters received crisis cards; non first-time suicide attempters received a phone call, and post-cards if the call could not be completed, or if the participant was in crisis and/or non-compliant with the post-discharge treatment. An As Treated strategy, accounting for the actual intervention received, was combined with subgroup analyses.

**Results:**

1,040 patients were recruited and randomized into two groups of N = 520, from which 53 withdrew participation; 15 were excluded after inclusion/exclusion criteria reassessment. AlgoS first attempters were less likely to reiterate suicide attempt (SA) than their TAU counterparts at 6 and 13–14 months (RR [95% CI]: 0.46 [0.25–0.85] and 0.50 [0.31–0.81] respectively). AlgoS non-first attempters had similar SA rates as their TAU counterparts at 6 and 13–14 months (RR [95% CI]: 0.84 [0.57–1.25] and 1.00 [0.73–1.37] respectively). SA rates were dissimilar within the AlgoS non-first attempter group.

**Conclusions:**

This new set of analysis suggests that crisis cards could be efficacious to prevent new SA attempts among first-time attempters, while phone calls were probably not significantly efficacious among multi-attempters. Importantly, phone calls were informative of new SA risk, thus a key component of future interventions.

## Introduction

With a toll nearing one million per year, suicide is the 17^th^ leading cause of death worldwide, and the 10^th^ cause of death for adults 18–40 years old [1,2]. Suicide prevention is now considered a global public-health priority, and large-scale multimodal programs are deemed the most efficient strategy to adopt [1]. Brief Contact Interventions (BCIs) in the immediate aftermath of suicide attempts (SAs) were identified as relevant pieces to integrate into such programs [3]. BCIs encompass a range of timely interventions designed to help patients in coping with prospective suicide crisis, notably by maintaining connection with the health care system. From a public health perspective, BCIs are particularly appropriate because (1) they target one of the most highly at-risk population, since SA is the main predictor for subsequent suicide behavior, with a maximum risk in the immediate aftermath of an index SA [2,4–7]; (2) rather than a substitute, they are intended to complement usual treatment and augment its effectiveness; and (3) as they can easily be initiated from emergency departments (EDs), they do not require on-site mental-health specialist, and are therefore easily scalable up to an entire population [8,9].

As shown by 3 recently published meta-analyses, results of efficacy trials of BCI are equivocal, however. Focusing on ED-based preventive interventions, Inagaki et al. found active contact and follow-up actions to significantly reduce the risk of suicide reattempt at 12 month (combined RR = 0.83, 95% CI: 0.71–0.98), but the effect was not maintained at 24 months (RR = 0.97, 95% CI: 0.76–1.22) [2]. Results obtained by Milner et al. are equally nuanced; when compared to control, the interventions resulted in significantly reduced number of suicide reattempts per person (incidence rate ratio IRR = 0.66, 95% CI:0.54–0.80), while the overall effects on suicide reattempt and suicide completion combined were not significant [10]. By contrast, Riblet et al. found that BCI were associated with significantly lower odds of suicide (OR = 0.20, 95% CI: 0.09–0.42)[3]; these interventions were WHO BCIs performed in low- and middle-income countries.

Results from AlgoS, a multi-modal intervention strategy that combines BCIs to fit participants’ profile through an algorithm, are in line with these meta-analyses [11]. AlgoS was built upon the hypothesis that each type of BCIs would not have an equal effect on all participants. Given the literature accumulated at that time, it assumed that the crisis card would be most efficient for first attempters, whereas multiple attempters would rather need a phone call. Post-cards would be used as a substitute if the participant was not reachable, and in complement to the phone call for most serious cases (non-adherent to the post-discharge treatment, and/or in suicidal crisis; see [9,11] and below for complete description). In the AlgoS trial, we indeed found the intervention to significantly reduce suicide reattempts or loss to follow-up at 6 months (19.5% vs. 25.1%, p = 0.034), contrasting with a non-significant reduction of suicide reattempts analyzed alone (12.8% vs. 17.2%, p = 0.056). It is noteworthy that those equivocal results were obtained from Intent to Treat analysis (ITT), while Per Protocol (PP) analyses yielded more consistent result on suicide reattempts alone (10.2% vs. 15.2%, p = 0.022) and on suicide reattempts and loss to follow-up combined (17.6% vs. 24.8%, p = 0.007). This contrast, together with the inconsistencies of the literature, raises questions about the appropriateness of relying only on ITT analyses for the evaluation of BCIs in general, and of the AlgoS protocol in particular. Indeed, the New England Journal of Medicine issued in 2016 a series of questions that researchers should ask when an ITT analysis finds a “non-significant” treatment difference [12]. Having in mind that the PP analysis of AlgoS trial was favorable to the intervention, four of these questions were particularly relevant to our case: “is there some indication of potential benefit”; “was the population appropriate”; “do subgroup analysis elicit positive signals”; and “can alternative analysis help”.

Both ITT and PP analyses of AlgoS trial indistinctly encompassed the global intervention, without disentangling the effects of each component, which yet substantially differed by patient’s suicidal status (first vs. non-first attempter), and were further composite for non-first attempters. Consequently, even if we assumed sufficient statistical power, the reasons for inconclusive results couldn’t be firmly decided between: (1) insufficient efficacy across the board; (2) inefficacy among a particular sub-group or a particular aspect of the multifaceted intervention; or (3) a combination of (1) and (2). Also, ITT analysis included some participants that shouldn’t have entered the protocol, because information given at inclusion turned out to be erroneous and corresponded to exclusion criteria after correction. These participants were high-end re-attempters (over 3 SA within 3 years); such a limit was set because AlgoS investigators considered that BCIs would be insufficiently efficacious on high-end suicidal cases–for them, more intensive care would be appropriate [4]. Therefore, their inclusion in the ITT analysis might have resulted in an artificial deflation of intervention efficacy. Furthermore, strict ITT framework does not allow for post-hoc subgroup analyses, hence prevent complete evaluation of some BCIs multiple benefits. For instance, the 10–21 days phone calls in AlgoS, primarily intended to reach out to multiple attempters and reduce their reattempt risk, also provided information about their clinical state, which could have a risk-assessment value *per se*. Only fine-grain analyses, beyond a strict ITT framework applied to AlgoS as a whole, would aim at eliciting such value. Finally, usual PP approach excludes patients that are “non-adherent”. By contrast, AT approach keep these patients in the analysis, but in a separate group, in order to evaluate the treatment effect among them. In our case “non-adherence” was peculiar: (1) it consisted in cross-over between distinct type of BCI, not between BCI and non-BCI groups; (2) it originated from the investigator (erroneous assessment of participant’s suicide attempt history).

For all the above reasons, we decided to reexamine AlgoS data, with a post-hoc As Treated (AT) approach, subgroup analyses, and exclusion of participants that turned out to be ineligible. The goal of this strategy was (1) to complement previous assessment of AlgoS efficacy in terms of reduction of SA reoccurrence, while taking into account the actual intervention received by the patients and, accordingly, patients’ suicidal characteristics; (2) to assess the risk-assessment value of AlgoS 10–21 days phone calls among multiple attempters. This strategy was undertaken with acknowledgment of flaws it may generate, which we discuss herein.

## Methods

The Algos trial was authorized by the French Ministry of Health, and approved by the Comité de Protection des Personne of Nord-Pas-de-Calais (Ethics Committee). It was registered with ClinicalTrials.gov (NCT01123174).

### General procedure

The AlgoS intervention was evaluated through a multicenter RCT conducted in 23 French Emergency Departments (EDs) and psychiatric crisis centers. Eligible participants were randomized to receive either treatment as usual (TAU group) or BCIs, nature of which was predefined by the AlgoS algorithm (AlgoS group). Randomization was performed with a 1:1 group ratio. All patients gave written informed consent before randomization, and were blinded to their group attribution. A complete description of the protocol has been published elsewhere [9]; we give here details that are relevant to the present analysis.

### Study participants

SA was defined after Silverman et al. nomenclature [13] as a “self-inflicted, potentially injurious behavior (self-injury or self-poisoning) with a nonfatal outcome for which there is evidence (either explicit or implicit) of intent to die” [13]. This definition was clarified to all emergency physicians. Adult patients (≥ 18 years old) were offered to participate in the study if they presented at the ED within 7 days of an SA, provided that their total number of SA was no more than 3 over the 3 past years. For follow-up purposes, they had to be reachable by phone for 14 months. Patients who were homeless or under legal guardianship were not invited to participate.

### Intervention

**Fig 1:**
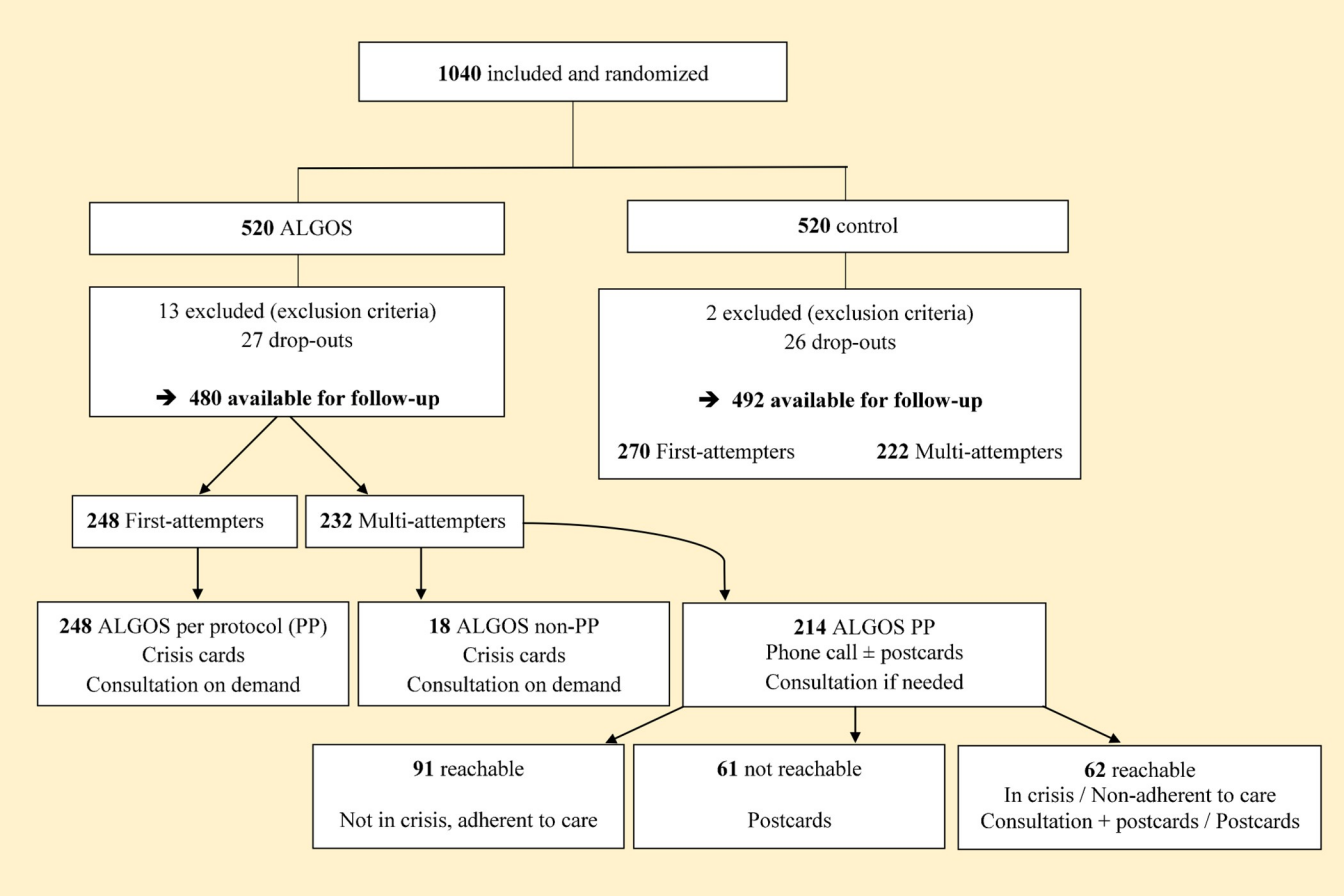
Study flowchart.

Participants of the TAU group received standard care for a SA as defined by the guidelines of the emergency center where the patients were attended. This included referral to outpatient clinics or ad-hoc post-crisis appointments. In accordance to AlgoS algorithm (Fig 1), patients assigned to the intervention group received the following intervention in addition to TAU:

- First-attempters: if the current SA was patient’s first lifetime SA, the patient received a crisis card at discharge, showing a toll-free number that could be called 24/7.
- Multi-attempters: if the current SA was not patient’s first SA, the patient received a phone call by a dedicated team of trained psychologists, on behalf of the initial attending team, between day 10 and day 21 after the SA. This phone contact aims were (1) to provide psychological support and asses patient’s mental health state, (2) to evaluate the patient’s adherence to post-discharge healthcare plan, and (3) to encourage the patient to make new contacts with his/her healthcare providers. At this point, the algorithm further split into 3 branches:
  ○ The patient could be reached, was neither in suicidal crisis nor in distress, and was adherent to the post-discharge treatment: no further intervention was undertaken (apart from TAU).
  ○ The patient could not be reached after 3 attempts at 3 different days and times: post-cards were sent at months 2, 3, 4 and 5.
  ○ The patient could be reached, but was in distress or suicidal crisis, and/or did not adhere to the post-discharge treatment: post-cards were sent at months 2, 3, 4 and 5; for patients in distress or suicidal crisis, an emergency consultation was set-up within 24 hours, at the center where they had been attended for their SA.

### Data collection

Baseline data were collected during a semi-structured ED visit led by an attending physician. They included demographics, basic information about the index SA (medication/drug overdose, use of alcohol), and information regarding current treatment for anxiety, depression, alcohol/drug related disorder, or eating disorder.

The outcome consisted in occurrence of at least one suicide reattempt during the follow-up period. Follow-up assessments were conducted at 6 months, and 13–14 months by a trained psychologist, blinded to patients’ group. The 13–14 month window for the second follow-up was chosen to avoid the 12-month SA anniversary date. To limit the recall bias, the patient status with respect to SA attempts was verified by cross-matching patients’ interview, reviews of medical records, and communication with patient’s GP, psychiatrists and caregivers.

### Data analysis

To complete and refine the results from the ITT assessment design of ALGOS, we performed post-hoc AT analysis and subgroup analysis on the same data set. Since the AT strategy privileges efficacy assessment as a function of actual intervention (or absence thereof) received by patients, groups were divided beyond the AlgoS vs. TAU dichotomy. Consequently, the plan of analysis was (1) to compare AlgoS first-attempters with their TAU counterparts; (2) to compare AlgoS multi-attempters with their TAU counterparts; then, in order to assess the 10–21 days phone call predictive value, AlgoS multi-attempters were compared by phone call outcome, using reachable and compliant patients as the reference group. AlgoS multi-attempters who received a crisis card instead of a phone call by mistake were also compared with reachable and compliant multi-attempters.

In line with the AT approach, patients who were unduly enrolled in the RCT were excluded post-hoc from analysis, in particular patients for whom the number of past SA was found post-randomization to exceed 3 in the 3 past years; AlgoS interventions are not expected to be efficacious on these patients.

The two outcomes were the existence of a SA reoccurrence within 6 months and within 13–14 months (binary yes/no). The main independent variable was the intervention actually received. Given the subgroups defined above, three sets of analyses were undertaken: (1) among firs-time attempters, crisis card and treatment as usual vs. treatment as usual alone (TAU); (2) among non first-time attempters, phone call and/or post cards and TAU vs. TAU alone; (3) within non first-time attempters assigned to AlgoS, comparisons by intervention received: crisis card, phone call alone (patient adherent to post-discharge treatment and not in crisis), post cards alone (patient non reachable), phone call and post cards (patient not adherent to post-discharge treatment and/or in crisis). The same sets of analyses were performed for baseline characteristics to elicit which of them could be significant confounders. We used chi-square tests with continuity correction or Fisher’s exact test to perform baseline comparisons. Six-month and 13-14-month new SA were analyzed by computing bivariate relative risks and their 95% confidence interval. Correction for confounding bias was then achieved by computing a multivariate logistic model with 6-months and 13-months new SA as outcome, and subgroups of intervention as a predictor. To identify potential confounders, variables that differed between groups at baseline with a p-value <0.15 were submitted to binomial logistic regressions. Variables were retained for the final model whenever the Wald Chi2 test reached the 0.10 level of significance. This model-building strategy complied with the Hosmer and Lemeshow guidelines [14]. All statistical analysis was done using the R statistical software version 3.3.2 [15].

## Results

### Study sample

**Fig 2:**
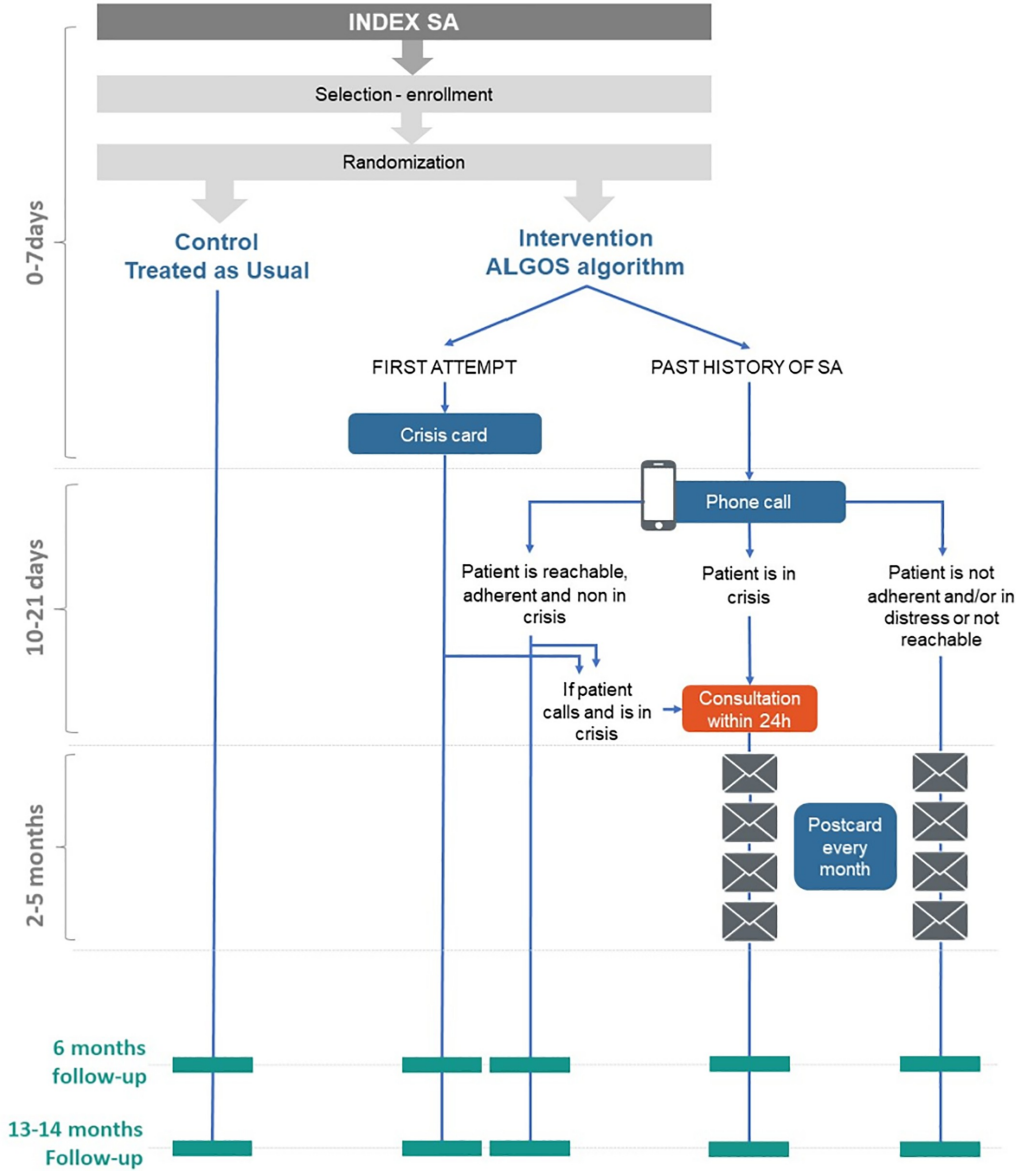
Analysis groups for the As Treated strategy. Number of participants (N) are those at baseline. N at 6-months and 13–14 months analysis are in Table 3.

The inclusion and analysis flow chart is presented on Fig 2. One thousand and forty patients were included and randomized into two groups of equal size (N = 520 each). Among them, 53 patients (27 in the AlgoS group, 26 in the control group) dropped out by consent withdraw, and 15 patients (13 in the AlgoS group, 2 in the control group) were excluded because they were reassessed with a history of more than 3 SA in the past 3 years. The final AT analysis thus included 480 individuals in the intervention group (248 first attempters and 232 multiple attempters) and 492 individuals in the TAU group (270 first attempters and 222 multiple attempters). Since AlgoS consisted in pro-active intervention originating from investigators, no member of the TAU group received it. In the AlgoS arm, the prescribed intervention was implemented among all 248 first-attempters, and among 214 out of 232 (92%) multiple attempters. The 18 multiple attempters who erroneously received a crisis card instead of a phone call were analyzed as a separate group, as per the AT strategy. The other 214 multiple attempters formed another group, which was further analyzed by phone call outcome (Fig 2).

### Baseline characteristics

Baseline comparisons are shown in Tables 1 and 2. AlgoS first-attempters did not differ from their TAU counterparts, except for SA by medication/drug overdose, which was slightly more frequent. AlgoS multi-attempters did not differ from their TAU counterparts. AlgoS multi-attempters subgroups differed on SA involving the use of alcohol, and on being treated or followed for alcohol/drug related disorders, which were more frequent among those not reachable by phone and those non-adherents or in crisis.

**Table 1:** Baseline characteristics of patients by group of analysis (part 1): First attempters vs. multi-attempters.

	First attempters			Multi-attempters		
	AlgoS N (*%)*	TAU N (*%)*	p *	AlgoS N (*%)*	TAU N (*%)*	p *
Age (years):						
18–35	125 (50)	126 (47)		81 (35)	86 (39)	
36–55	105 (42)	116 (43)	.401	116 (50)	110 (50)	.492
> 55	18 (8)	28 (10)		35 (15)	26 (12)	
Gender: men (vs. women)	97 (39)	115 (43)	.421	78 (34)	64 (29)	.270
Living alone (vs. in couple)	135 (54)	143 (53)	.737	118 (51)	119 (54)	.556
Employed (vs. unemployed)	172 (70)	182 (68)	.673	130 (56)	135 (61)	.328
SA by medication overdose	235 (95)	242 (90)	.030	221 (95)	214 (96)	.642
SA with alcohol	97 (39)	113 (42)	.776	109 (47)	98 (44)	.788
Treated or followed for:						
Depressive disorder	83 (34)	90 (34)	.996	121 (52)	118 (53)	.829
Anxiety disorder	101 (41)	109 (41)	.988	121 (52)	124 (57)	.367
Alcohol/drug related disorder	29 (12)	36 (13)	.564	59 (26)	42 (19)	.111
Eating disorder	7 (3)	6 (2)	.782	16 (7)	18 (8)	.623

*chi-square test if no more than 20% of the expected frequencies are less than 5 and none is less than 1, Fisher’s exact test otherwise

Abbreviations: TAU = Treatment As Usual, SA = suicide attempt.

**Table 2:** Baseline characteristics of patients by group of analysis (part 2): Actual intervention among multi-attempters.

	Multi-attempters				
	Phone call			Crisis card	p-value *
	Adherent, not in crisis N (*%)*	Not reachable N (*%)*	Non-adherent, or in crisis N (*%)*	N (*%)*	
Age (years):					
18–35	35 (38)	20 (33)	15 (24)	11 (61)	
36–55	43 (47)	33 (54)	34 (55)	6 (33)	.112
> 55	13 (14)	8 (13)	13 (21)	1 (6)	
Gender: men (vs. women)	29 (32)	24 (39)	18 (29)	7 (39)	.609
Living alone (vs. in couple)	42 (46)	33 (54)	36 (59)	7 (39)	.294
Employed (vs. unemployed)	55 (60)	32 (52)	29 (48)	14 (78)	.099
SA by medication overdose	86 (95)	56 (92)	61 (98)	18 (100)	.265
SA with alcohol	34 (37)	36 (59)	33 (53)	6 (33)	.027
Treated or followed for:					
Depressive disorder	44 (48)	32 (52)	38 (61)	7 (41)	.331
Anxiety disorder	50 (55)	26 (43)	33 (53)	12 (67)	.296
Alcohol/drug related disorder	15 (17)	23 (38)	19 (31)	2 (11)	.010
Eating disorder	7 (8)	4 (7)	4 (6)	1 (6)	.976

*chi-square test if no more than 20% of the expected frequencies are less than 5 and none is less than 1, Fisher’s exact test otherwise

Abbreviations: SA = suicide attempt.

### AlgoS efficacy on SA attempts

In the bivariate analysis (Table 3), AlgoS first-attempters had significantly lower risk of SA attempts than their TAU counterparts both at 6 months and 13–14 months (RR = 0.46, 95% CI: 0.25–0.85, and RR = 0.50, 95% CI: 0.31–0.81, respectively). AlgoS multi-attempters did not differ significantly from their TAU counterparts, either at 6 months or at 13–14 months.

**Table 3:** Suicide reattempts at 6 months and 13–14 months follow-ups: Rates (per 100) and corresponding relative risks.

	6 months follow-up			13–14 months follow-up		
	N	suicide reattempt (per 100)	RR [95% CI]	N	suicide reattempt (per 100)	RR [95% CI]
First attempters						
TAU	241	13.3	ref.	215	19.5	ref.
AlgoS	227	6.2	0.46 [0.25–0.85]	215	9.8	0.50 [0.31–0.81]
Multi-attempters, AlgoS vs. TAU						
TAU	204	21.0	ref.	187	28.3	ref.
AlgoS	219	17.8	0.84 [0.57–1.25]	201	28.3	1.00 [0.73–1.37]
Multi-attempters, within AlgoS						
Phone call and its outcome						
Adherent, not in crisis → no more action	85	12.9	ref.	74	23.0	ref.
Non reachable → post cards	57	12.3	0.95 [0.39–2.30]	52	17.3	0.75 [0.36–1.56]
Non-adherent / in crisis → post cards	59	25.4	1.96 [0.97–3.97]	57	42.1	1.83 [1.09–3.07]
Crisis card	18	33.3	2.58 [1.10–6.06]	18	38.9	1.69 [0.82–3.45]

Abbreviations: TAU = Treatment As Usual

Since significant results shown in Table 3 could be the results of differences between groups at baseline characteristics (Tables 1 and 2), we performed logistic regressions (LR) to adjust for potential confounders (Tables 4 and 5). SA attempts at 6 and at 13–14 months were the dependent variables (two distinct sets of LR), AT intervention groups were the independent variables as shown in Tables 1 and 2, and baseline variables from Tables 1 and 2 with p<0.15 were potential confounders. These confounders were kept in final models if their corresponding p-value were below 0.10 in the LR, and dropped otherwise (Table 4). Results of final LR, detailed in Table 5, were in line with results of bivariate analyses: AlgoS first-attempters had significantly lower odds of SA attempts than their TAU counterparts, at 6 months and 13–14 months follow-ups (no adjustments needed).

**Table 4:** Logistic regressions (LR) of suicide reattempt at 6 and 13–14 months by As Treated (AT) group or subgroup: Selection of baseline variables as potential confounders.

		6 months follow-up		13–14 months follow-up	
Main independent variable: AT group or subgroup	Other independent variables: potential confounders	p-value initial LR	Included (I) / Excluded (E) in final LR	p-value initial LR	Included (I) / Excluded (E) in final LR
**First attempters,** **AlgoS (ref: TAU)**	SA by medication overdose	.401	E	.446	E
**Multi-attempters, within AlgoS subgroup** * (ref: phone call, patient is adherent and not in crisis)	Age	.603	E	.560	E
	Employed	.567	E	.296	E
	SA with alcohol	.035	I	.151	E
	Treated/Followed for alcohol/drug disorder	.017	I	.001	I

* Subgroups are

(1) phone call, patient is adherent and not in crisis → no more action

(2) phone call, patient is not reachable → post cards

(3) phone call, patient is not adherent and/or in crisis → post cards (and consultation if in crisis)

(4) crisis card (instead of a phone call)

**Table 5:** Logistic regressions (LR) of suicide reattempt at 6 and 13–14 months by As Treated (AT) group or subgroup: Odds-ratios of SA reattempt adjusted for potential confounders.

	6 months follow-up		13–14 months follow-up	
Main independent variable: AT group or subgroup	OR	95% CI	OR	95% CI
First attempters				
TAU	1 (ref.)		1 (ref.)	
AlgoS	0.43	0.22–0.83	0.45	0.25–0.78
Multi-attempters, within AlgoS				
Phone call: Adherent, not in crisis → no more action	1 (ref.)		1 (ref.)	
Phone call: Non reachable → post cards	0.56	0.19–1.65	0.49	0.19–1.32
Phone call:Non-adherent / in crisis → post cards	1.59	0.64–4.01	2.23	1.01–4.94
Crisis card	3.97	1.16–13.6	2.51	0.81–7.73

### Risk assessment value of the 10–21 days phone call

AlgoS multi-attempter subgroup bivariate analysis showed that, with reference to participants who were reachable, adherents, and not in crisis: (1) those not reachable did not significantly differ; (2) those non-compliant and/or those in crisis had significantly higher risk of SA attempts at 13–14 months (RR = 1.83, 95% CI 1.09–3.07); (3) those who received a crisis card instead of a phone call had significantly higher risk of SA attempts at 6 months (RR = 2.58, 95% CI: 1.10–6.06). These results did not change substantially after adjusting for potential confounders in multivariate analysis (Table 5): those non-compliant and/or those in crisis had higher odds of SA attempts, with corresponding OR significantly different from 1 at 13–14 months; and those receiving a crisis card had higher odds of SA attempts, with corresponding OR significantly different from 1 at 6 months.

## Discussion

### Significance of findings

The post-hoc Analyses showed a significant risk reduction of SA attempts among first-time attempters by delivery of crisis cards thus adding to results found by Evans et al. [16,17]. According to relative risk 95% confidence intervals, risk reduction might range from one fifth in the worst-case scenario to three quarters in the best-case scenario. Crisis card delivery is an easy, low-cost and relatively safe intervention to implement. We believe that the present results reduce the literature ambiguities and invite considering the scale-up of such minimal action for first attempters to a population-size prevention strategy. Alternatively, due to unknown bias introduced by subgroup and AT analyses, one might want to wait for further research results before embarking on such scaling-up. In addition, it might be challenging in an ED environment to ascertain whether an individual has actually no past history of suicide attempt. Therefore, future research on the efficacy of crisis card in the multi-attempters population, probably as a supplement to other interventions, is needed; if proved efficacious, crisis card delivery could then be proposed as a basic intervention towards any type of suicide attempter.

By contrast, phone calls, possibly complemented with or substituted by post-cards, did not show significant reduction of SA attempts among multi-attempters. Although neither lack of power nor bias can be ruled out (AlgoS was powered to be evaluated as a whole, not by subgroups), those negative results are coherent with a recent meta-analysis [18] and provide a plausible explanation for the inconclusive overall effect of AlgoS found with the ITT analysis. A possible reason for such insufficient efficacy is the modesty of the intervention: only one phone call (over three attempts) in the immediate aftermath of the SA. Likewise, the number of post-card sent was small, and the efficacy of post-cards is still equivoque in the literature [4,16,19–21]. In other studies, multiple attempters were found to show higher intentionality in their attempts, higher levels of psychopathology, and higher risk of reattempt than first-time attempters [22,23].

Phone calls had a risk-assessment utility, since their outcome was predictive of SA reattempt: participants found to be in crisis and/or non-compliant were more likely to reiterate a SA than their compliant and not-in-crisis counterparts. In spite of their apparent insufficient efficacy, phone calls could therefore be kept within a multimodal strategy, as a surveillance component, results of which could trigger more aggressive actions–in AlgoS, subsequent actions were limited to sending post-cards.

The current literature does not give unequivocal directions about how to optimize surveillance systems for multiple attempters. Heterogeneity might be one of the most prominent characteristic of suicidal populations, and pathways to SA are highly complex [4,24–26]. There is a growing recognition that suicide prevention needs a comprehensive, systemic approach, involving a complex set of interventions that are likely to complement each other [24–29], even in the face of underwhelming results (for example [6]). Consequently, AlgoS’ multiple components should be further developed into a finer-grain algorithm. Depending on the outcome of short-term clinical assessments, interventions could be diversified in nature, frequency, and intensity. In addition, BCIs could incorporate new technologies such as mobile phone text messages [30], emails, or social media, in complement to traditional postcards or phone calls.

### Study strengths and limitations

Our analysis gave priority to the intervention actually received by patients, and performed post-hoc subgroups analysis, thus unveiling aspects of the intervention that would otherwise stay obliterated. It found efficacy discrepancies between subgroup of patients and, in turn, informed aspects of the overall intervention that could be improved or changed. We must acknowledge, however, that exclusion of participants who were erroneously included can create bias and generate misleading results, and the same is true for the AT approach [12]. If we call ITTe the intervention effect that is estimated with ITT rules, all randomized subjects ought to be analyzed, whether rightly or wrongly included. Indeed, ITTe is a good estimate of the intervention effectiveness, because in real world erroneous treatment assignment occurs, as well as imperfect adherence to treatment. In scenarios where the true treatment effect is null or small, ITTe is also the least biased estimate of efficacy; in general, ITTe provides a more conservative estimate of treatment efficacy, and therefore will avoid overstating an effect when one doesn't exist. But when the true effect is moderate or large, however, ITTe turns out to be the most biased estimate of treatment efficacy, towards the null [31–33], hence with a tendency of finding an absence of effect when one exists. Nevertheless, one must bear in mind that, depending on the real unknown magnitude of the intervention effect, and depending on quantitative and qualitative characteristics of all departures from the protocol, an AT estimate of efficacy can also be biased, most likely overinflated. Our results, then, should be regarded as optimistic.

By the very nature of the intervention among multi-attempters, based on the result of a phone call that was made for the AlgoS group only, subgroup AlgoS vs. TAU comparisons were not possible. We made subgroup comparisons within the AlgoS group, taking the group with the fewest expected SA attempts as reference, in order to elicit subgroup differences that could unveil the possible risk-assessment value of the phone call outcome. This outcome is very likely to correspond to unmeasured psychopathology. Indeed, it can be hypothesized that presenting with severe mental health problems increases the risk of suicide reattempt and the probability of being found unreachable or in crisis at 10–21 days. Rigorously collecting psychopathology, however, is labor intensive and requires psychiatric expertise, both of which are barely compatible with the routine activity of an ED physician. As far as AlgoS is meant to be scaled up to an entire population, where availability of psychiatric time and expertise is not ubiquitous, such assessment can only be deferred after the day 10–21 call, let alone assessment inaccuracies in the wake of a suicidal crisis. Indeed, algorithm-based BCIs have to compose with uncertainties, and rely on clinical proxies; in view of our results, the outcome of the 10–21 days call can be one of these proxies.

### Conclusions

This study suggests that crisis cards (green cards) might be a useful tool to prevent SA attempts among first-time attempters, and that phone calls are unlikely to be efficacious among multi-attempters, but are informative of SA reattempt risk. We believe that a more elaborated and vigorous BCI, capitalizing on the results of the current analyses, deserve investigation.

## Supporting information

S1 File Dataset. Dataset containing all variables used to produce results of this article.
(XLSX)
